# Anaemia among Students of Rural China's Elementary Schools: Prevalence and Correlates in Ningxia and Qinghai's Poor Counties

**DOI:** 10.3329/jhpn.v29i5.8901

**Published:** 2011-10

**Authors:** Renfu Luo, Linxiu Zhang, Chengfang Liu, Qiran Zhao, Yaojiang Shi, Grant Miller, Elaine Yu, Brian Sharbono, Alexis Medina, Scott Rozelle, Reynaldo Martorell

**Affiliations:** ^1^Center for Chinese Agricultural Policy, Institute of Geographical Sciences and Natural Resources Research, CAS, Beijing 100101, China; ^2^School of Economic Management, Northwest University, Xi'an, China; ^3^Shorenstein Asia Pacific Research Center, Freeman Spogli Institute, Stanford University, Stanford, California, USA; ^4^Rollins School of Public Health, Emory University, Atlanta, Georgia, USA

**Keywords:** Anaemia, Anaemia, Iron-deficiency, Cross-sectional studies, Educational performance, Primary school students, China

## Abstract

Although the past few decades have seen rising incomes and increased government attention to rural development, many children in rural China still lack regular access to micronutrient-rich diets. Insufficient diets and poor knowledge of nutrition among the poor result in nutritional problems, including iron-deficiency anaemia, which adversely affect attention and learning of students in school. Little research has been conducted in China documenting the prevalence of nutritional problems among vulnerable populations, such as school-age children, in rural areas. The absence of programmes to combat anaemia among students might be interpreted as a sign that the Government does not recognize its severity. The goals of this paper were to measure the prevalence of anaemia among school-age children in poor regions of Qinghai and Ningxia, to identify individual-, householdand school-based factors that correlate with anaemia in this region, and to report on the correlation between the anaemic status and the physical, psychological and cognitive outcomes. The results of a cross-sectional survey are reported here. The survey involved over 4,000 fourth and fifth grade students from 76 randomly-selected elementary schools in 10 poor counties in rural Qinghai province and Ningxia Hui Autonomous Region, located in the northwest region of China. Data were collected using a structured questionnaire and standardized tests. Trained professional nurses administered haemoglobin (Hb) tests (using Hemocue finger prick kits) and measured heights and weights of children. The baseline data showed that the overall anaemia rate was 24.9%, using the World Health Organization's blood Hb cut-offs of 120 g/L for children aged 12 years and older and 115 g/L for children aged 11 years and under. Children who lived and ate at school had higher rates of anaemia, as did children whose parents worked in farms or were away from home. Children with parents who had lower levels of education were more likely to be anaemic. The anaemic status correlated with the adverse physical, cognitive and psychological outcomes among the students. Such findings are consistent with findings of other recent studies in poor, northwest areas of China and led to conclude that anaemia remains a serious health problem among children in parts of China.

## INTRODUCTION

Iron-deficiency anaemia is the most common nutritional deficiency worldwide, affecting approximately a quarter of the global population and is particularly widespread in developing countries (1,2). Deficiency in iron impedes the transport of oxygen between the brain and body, carried by haemoglobin (Hb) protein in red blood cells. In severe cases, anaemia can be life-threatening due to loss of blood or heart failure. Many vital aspects of human health are adversely affected by anaemia, including energy, temperature regulation, behaviour, and immune function (1,3). Furthermore, numerous studies have linked less extreme iron deficiency and anaemia to cognitive impairment and altered brain function (2,4).

Beyond immediate and long-term health consequences, anaemia is also associated with negative effects on learning abilities. Literature over the past three decades show links between iron deficiency (particularly during early childhood) and poor cognitive performance (5) and motor (6) and psychomotor development (7). Consequently, negative correlations have been identified between childhood anaemia and academic achievement, including grades, attendance, and attainment (8-11). Recognizing and treating anaemia are especially critical since developmental and behavioural damages have been shown to have long-lasting effects into adulthood in animal studies of biological mechanisms (12,13) and also in human studies (14-17). Due to the long-term effects, anaemia may hinder children's economic and social mobility, which perpetuates inequality trends between rural and urban regions (11).

The prevalence of anaemia generally decreases as incomes rise according to the World Health Organization's (WHO's) global database on anaemia and other international research and experiences (1,18). Yet paradoxically, China appears to deviate from the observed trends. Incomes in China have dramatically risen over the past three decades, even in rural areas. Despite greater wealth and growing government commitment for quality education, a recent country-wide 2002 China Nutrition and Health Survey found that 20.8% of residents in rural China are so deficient in iron as to be diagnosed as anaemic (19). Results of other available province-specific studies indicate even a higher prevalence of anaemia among rural Chinese children: 40% of school children in Gansu province and 50-60% of students in Guizhou province (20,21). While regional studies generally include small sample populations and weak (reported) methodology, results still corroborate the prevalence of anaemia in rural areas high enough to be classified as either a moderate or severe public-health concern by the WHO standards (22). In fact, this study builds on the work of a previous survey in Shaanxi province in 2008, using nearly identical sampling and assessment methods as this study (23). The Shaanxi study found a 39% prevalence rate of anaemia among fourth and fifth grade students, just shy of the 40% anaemia cut-off required by the WHO to be considered a severe public-health concern. Addressing iron deficiency is a priority given its serious health and education effects and its implications for inequality. Distribution of income in China has been highly unequal in the recent past (24). The prevalence of high rates of anaemia suggests that the socioeconomic differences persist in economic and health conditions in China, despite the Government's efforts to reduce inequality.

In this paper, we have three objectives. First, we want to increase our understanding of the anaemia problem in the poor areas of Ningxia and Qinghai by identifying the prevalence rates of anaemia in the survey areas. Second, we hope to identify the individual-, householdand school-based factors that may be correlated with anaemia status. Finally, building on recent efforts (25), we aimed to further our understanding of the health and educational impacts of anaemia by describing correlations between the anaemia status and the performance of students on tests of the physical, psychological and cognitive outcomes. Our results are based on a large survey of over 4,000 fourth and fifth grade students, mostly aged 9-12 years, from 76 rural elementary schools in 10 of the poorest counties in Qinghai province and Ningxia Hui Autonomous Region, both of which are located in the poor, northwest region of China.

## MATERIALS AND METHODS

### Defining anaemia

The initial step is to address how anaemia is measured and defined before presenting data. Normal Hb distribution is known to vary according to both age and altitude. First, the age range of our sample population results in some uncertainty about what Hb cut-off should be used for reporting the anaemia levels. Since we focused on fourth and fifth graders in the sample population, the large majority (76.8%) of the students were aged 9-12 years. The WHO recommends an Hb level cut-off of 115 g/L for children aged 5-11 years and 120 g/L for children aged 12-14 years (22). The large majority (76.8%) of our sample subjects were aged 9-12 years. In this paper, we calculated the anaemia rates based on the cut-off of 115 g/L for children aged 11 years and under and the cut-off of 120 g/L for children aged 12 years and over.

Second, it is important to note that, at higher altitudes, Hb levels rise to compensate for the lower partial pressure of oxygen and reduced oxygen saturation of blood. Increases in red cell production ensure that sufficient oxygen is supplied to body tissues at elevations of over 1,000 metres (26). Although a population at a high altitude will reveal a higher density of Hb, delivery of functional oxygen to the body and brain is equivalent to a sea-level population with a lower Hb density (27). Thresholds for anaemia are calculated for populations living at sea-level. Therefore, to compare the anaemia status between populations living at varying elevations, the Hb density of populations at high altitudes must be adjusted downward to account for the reduced saturation of blood, thus giving their effective Hb density in terms of Hb levels at sea-level. [This has the equivalent effect of increasing the threshold density to assess the anaemia status of someone at a greater elevation]. Adjustments can range in size from a single g/L to several tens, depending on the altitude.

All the students in our study sample attend schools at altitudes of above 1,000 metres; therefore, their raw Hb measurements must be adjusted for altitude to be considered accurate (26). To compare the Hb levels in our sample to sea-level-based Hb density thresholds, we adjusted reported Hb counts based on the altitude of each student's school, using the following curvilinear equation developed by the Pediatric Nutrition Surveillance System at the U.S. Centers for Disease Control and Prevention (U.S. CDC). This equation has been tried and tested for altitudes between 1,200 and 3,000 metres (28). The adjustment of U.S. CDC has been widely used by a number of other studies, including Zimmerman *et al*. (29). The formula is:

Hb_adjustment_ = −0.32 * (altitude in metre * 0.0033) + 0.22 * (altitude in metre * 0.0033)

The raw Hb levels and the average altitude by county can be found in Appendix Table 1.

### Data

Studentand school-level data were collected about fourth and fifth grade elementary school students from 76 rural primary schools in Qinghai and Ningxia counties during October 2009. Conducting the study in Qinghai and Ningxia is advantageous since the sample population represents over 11.5 million people and includes two distinctly separate geographic regions of western China. Ningxia primarily consists of arid, dry desert while Qinghai is mountainous, bordering the Tibetan Plateau, with an average elevation of 3,000 feet above sea-level. Based on income per capita, both are among the poorest provinces in China. In Ningxia, the average per-capita income is RMB 3,180, 23% below the mean national income (30). Across Qinghai, the average per-capita income is even lower (RMB 2,683), 35% below the mean national income (30).

Conducting the study in Ningxia and Qinghai has another benefit. Specifically, by working in these provinces, it makes it possible to consider this paper as an extension of Luo *et al*. (23), which looks at anaemia in Shaanxi province, another poor province in northwest China. In this paper, the anaemia rate has been shown as 38%. Qinghai and Ningxia are also poor provinces in western China. Therefore, one of the contributions of this paper is to test the representativeness of the findings from Shaanxi. If the findings are similar, the results in this paper could be representative of a problem that affects 100-200 million poor rural people in western poor provinces of China.

**Fig. 1.**
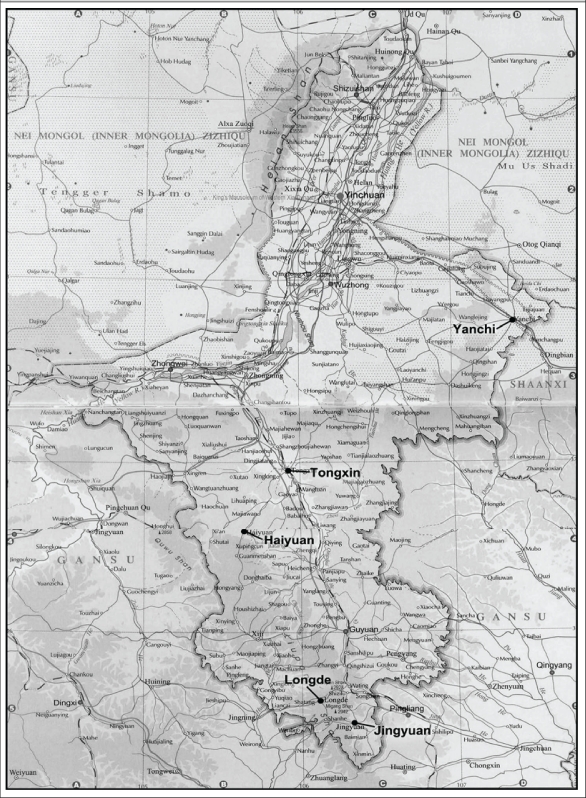
Map of sample counties in Ningxia

**Fig. 2.**
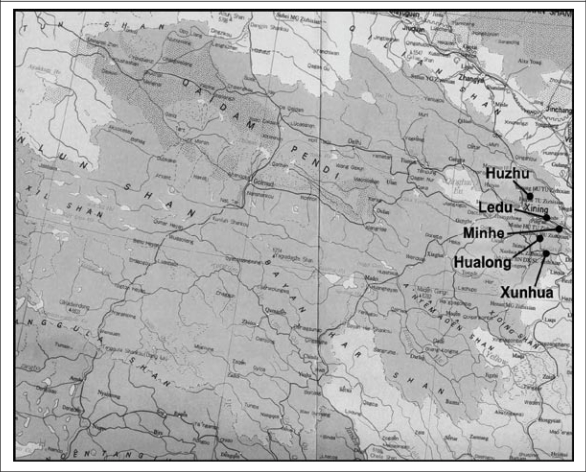
Map of sample counties in Qinghai

Our sample-selection procedure employed a randomized sampling approach. In choosing our sample, we first obtained a list of all counties in each of the two regions and assigned each county on the list a poverty ranking according to the per-capita income of each county in 2008. Once each county was assigned a poverty indicator, we randomly chose 10 poor counties from among the 30 poorest counties in Qinghai and Ningxia. Of these, five were in Qinghai, and five were in Ningxia. [In the selection of sample counties in Qinghai, we focused on the agricultural regions in the eastern part of the province where about 70% of the total population lives. Qinghai generally consists of two regions—agricultural and pastoral—, which are mostly part of the Tibetan plateau]. The location of the study counties is shown in Figure 1 and 2.

Using the official records, we next created a sampling universe of all primary schools in the 10 sample counties. In total, 643 schools were identified. We then used a combination of official records and a canvass survey to identify all schools with the following characteristics: (a) six grades (i.e. complete primary schools, or wanxiao), (b) boarding facilities, and (c) 400 or more students. These criteria were used because the Government of China is currently consolidating the existing rural schools into new ones with these characteristics. We identified 76 schools that met these criteria. There are, on average, 114 fourth and fifth grade students per school. The survey was conducted in all the 76 schools, data on which were summarized and grouped by county in Table 1, Panel 1. Our sample is representative of school-age children in poor, rural areas of Qinghai and Ningxia.

With the assistance of nursing teams from the Xi'an Jiaotong Medical School, we collected socioeconomic information and also information on Hb concentrations from half of all the fourth and fifth grade students (n=4,130) in the 76 study schools. The dataset was gathered by six teams of six enumerators. In each team, one person collected data on the school from the principal and the school's fourth and fifth grade homeroom teachers while three collected individual and household socioeconomic information from the students. Two trained nurses carried out the Hb testing and measured heights and weights of the children. Information on age was taken from the birth records that are part of each student's matriculation folder, widely considered to be accurate. Hb levels were measured onsite using a Hemocue Hb 201+ system.

The first part of the student survey collected data on basic socioeconomic information about each student. The survey questions included details about the student's gender, age, and family structure. They also asked about where the student lived—at home or in the school's boarding dormitories. Each student also took a form home to his/her parents who filled out information on their educational background, age, and occupation.

The second part of the survey consisted of a series of tests designed to measure the effects of anaemia on the cognitive development of the students. Two tests were administered: a standardized mathematics test and a psychological test. The mathematics test was based on a sub-set of a test originally created for the Trends in International Mathematics and Science Study (TIMSS). The psychological test—the Mental Health Test (MHT)—was developed by Professor Bucheng Zhou (31). The Zhou MHT contains 100 yes/no questions. The lower test scores correspond to a healthier mental state. The MHT has a reliability of 0.84-0.88 and a retest reliability of 0.78-0.86.

### Statistics

To make sense of our prevalence data, we adopted two distinct statistical methodologies. First, we presented a simple breakdown of altitude-adjusted Hb levels and the anaemia rates (using age-specific anaemia cut-offs) by region. Next, we used multivariate probit models with county dummy variables and school dummy variables to more rigorously assess regional variability in the prevalence of anaemia.

To identify the individual-, householdand school-based factors associated with anaemia status, we first presented descriptive statistics on anaemia rates for each potential correlate. Next, we conducted a multivariate linear regression to identify the relationship between each potential correlate and Hb levels.

To achieve our final objective of showing the relationship between the anaemia status and the measurable outcomes, we first presented a simple comparison of anaemic and non-anaemic students in terms of their physical measurements and their scores on a series of psychological and cognitive tests. We then ran a series of multivariate regressions, using the dependent variable as height-for-age z-score, a dummy variable equal to one if the student was stunted, body mass index (BMI) z-score, score on a standardized mathematics examination and score on a psychological examination and controlling for a number of individualand household-based characteristics.

**Table 1.** Distribution of sample schools, students, and anaemia rates across counties

Panel 1. Distribution of sample schools and students			
Item	No. of schools	No. of students	% of students
Total sample	76	4,130	100
Qinghai	39	1,474	35.7
Ningxia	37	2,656	64.3
Qinghai province			
Xunhua ( 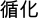 )	10	463	11.2
Ledu ( 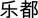 )	3	126	3.1
Hualong ( 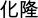 )	6	247	6.0
Huzhu ( 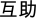 )	10	286	6.9
Minhe ( 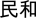 )	10	352	8.5
Ningxia province			
Yanchi ( 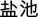 )	5	410	9.9
Tongxin ( 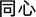 )	11	683	16.5
Haiyuan ( 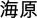 )	12	852	20.6
Longde ( 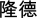 )	6	508	12.3
Jingyuan ( 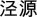 )	3	203	4.9
Female		1.987	48.1
Male		2.143	51.9
Panel 2. Anaemia prevalence rates, by region			
Item	Adjusted haemo-globin level (g/L) *	No. of students with anaemia	% of students with anaemia
Total sample	125.2	1,027	24.9 (27.3)
Qinghai	118.9	603	40.9
Ningxia	128.7	424	16.0
Qinghai province			
Xunhua ( 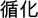 )	108.3	349	75.4
Ledu ( 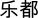 )	127.5	18	14.3
Hualong ( 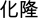 )	117.9	94	38.1
Huzhu ( 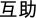 )	122.6	75	26.2
Minhe ( 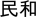 )	127.5	67	19.0
Ningxia province			
Yanchi ( 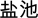 )	125.6	77	18.8
Tongxin ( 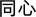 )	124.6	160	23.4
Haiyuan ( 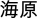 )	132.7	70	8.2
Longde ( 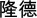 )	130.9	71	14.0
Jingyuan ( 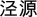 )	125.9	46	22.7

*Haemoglobin counts were adjusted to account for altitude effects according to calculations by U.S. CDC (28). See Appendix Table 1 for further details on the adjusted values. The WHO recommends a haemoglobin level cut-off of 115 g/L for children aged 5-11 years and that of 120 g/L for children aged 12-14 years. The majority of the sample population was aged 9-12 years. In this table, the anaemia rates were calculated based on the cut-off of 115 g/L for children aged 11 years and under and that of 120 g/L for children aged 12 years and over. The 24.9% figure represents the average anaemia rate based on weights of the study sample. We also reported the average anaemia rate by weighting based on the official statistics on the number of primary school students in rural Qinghai and Ningxia in 2008.

CDC=Centers for Disease Control and Prevention;

WHO=World Health Organization

The findings of these analyses are presented in the Results section.

### Ethical approval

In designing and implementing the study, we paid close attention to the ethical concerns that arise when testing children for medical conditions. Our project obtained ethical approval from the Institutional Review Board (IRB) of the Stanford University. In accordance with the IRB requirements, the study children provided oral assent for the project while the school principals—who are the children's legal guardians while the children are in school—provided their consent. All students involved in the project (whether anaemic or not) received letters informing their parents of their Hb status, along with information about the health consequences of anaemia and how to prevent and treat it.

Students with severe anaemia, defined by Hb levels of lower than 70 g/L (26), were immediately sent to receive treatment at the local health centre. All other students found to be anaemic received treatment in the classroom.

## RESULTS

### Prevalence of anaemia in Qinghai and Ningxia

Despite the growing wealth in China, our results indicate that anaemia is still prevalent among the sample students in Qinghai and Ningxia. Across all the schools surveyed, the average Hb level was 125.2 g/L, after adjusting for altitude. The Hb levels were normally distributed with a standard deviation of 14.5. Using the age-specific anaemia cut-offs, we calculated that 1,027 (24.9%) of the 4,130 students surveyed were anaemic (Table 1, Panel 2).

The overall prevalence of anaemia disguises geographical variation across counties. To gain a clearer picture of this variation, we conducted a multivariate probit regression of county dummies on anaemia rates using the following specification:

Y_ij_ = a_0_ + a_1_ * Countydummy_j_ + e_ij_ [1]

where *Y_ij_* is a dummy variable equal to one if student *i* in county *j* is anaemic; Countydummy_j_ represents a set of nine dummy variables that are equal to one if student *i* is in county *j*; and *e_ij_* is an error term that correlated within counties.

Although, for the sake of brevity, the full results of this model are not presented here, the p value of the test (an F-test of the joint significance of the dummies) showed a significant county effect (p<0.001). Haiyuan county in Ningxia had the lowest average anaemia rate (8.2%), and Xunhua county in Qinghai had the highest average anaemia rate (75.4%).

Beyond the variation observed among the counties, we also conducted a multivariate regression of school dummies on anaemia rates to determine whether the school-level variation was a significant factor. The model specification was identical to that used in equation [1] but substituting a set of 75 school dummy variables for the county dummies. The p value of the test showed a significant school effect (p<0.001), indicating a significant variation across the schools. The anaemia rate ranged from 5.9% to 100%.

### Individual, household and school-based correlates of anaemia

In considering which factors may correlate with anaemia status, we identified three general categories: individual-based factors, such as age and gender; household-based factors, such as parental education and occupation; and school-based factors, such as whether a student boards or eats at school. In this section, we first considered each factor separately. In the second part of the section, we conducted a multivariate analysis to determine which factors were significantly associated with anaemia status.

In the first category, males and females did not appear to differ in their anaemia rates, although females were slightly more likely to be anaemic (Table 2). Pre-pubescent children were unlikely to show the large gender differences common during adolescence and adulthood, when females were more likely to be iron-deficient due to blood loss during menstruation (32). Younger children had lower rates of anaemia, and these rates increased with the increase in the age of children (Table 2).

Household characteristics that we identified as being potentially correlated with anaemia included parental education and parental employment. In terms of employment, rural parents may work full-time in the farm (full-time farmer), part-time in the farm, and part-time in an off-farm job (part-time farmer), or full-time off the farm (off-farm worker). The large migrant labour force of China's also means that some rural parents may be working in a different town, county, or province. We attempted to assess the parental migrant status by asking whether the parent lived at home for most of the year, or was away from home for most of the year.

Some household characteristics also correlated with anaemia (Table 2). For example, higher levels of parental education were associated with lower anaemia rates. A number of demographic studies support this result (33). However, the causal relationship has yet to be defined (33-35).

**Table 2.** Individual-, householdand schoolbased characteristics that may correlate with anaemia

Charact-eristics	% with anaemia	% who boarded	% of total students
Gender			
Female	24.6	37.5	48.1
Male	25.5	39.6	51.9
Age-group (years)			
<9	21.6	29.7	38.1
9-10	24.8	37.9	27.9
10-11	27.1	46.9	21.6
>11	30.2	51.7	12.7
Education of fathers			
Illiterate	28.8	44.1	20.1
Primary school	26.4	42.4	41.3
Junior high school	22.1	33.6	29.3
High school	19.8	25.7	8.2
College or above	18.9	18.9	1.1
Education of mothers			
Illiterate	27.3	41.8	48.3
Primary school	24.2	39.9	34.8
Junior high school	19.8	27.4	13.7
High school or above	17.2	21.5	3.2
Employment of fathers			
Full-time farmer	27.5	43.5	33.3
Part-time farmer	24.8	37.9	50.0
Off-farm worker	21.2	30.7	16.7
Employment of mothers			
Full-time farmer	24.9	41.2	51.3
Part-time farmer	24.6	38.1	38.1
Off-farm worker	25.3	27.2	10.6
Residence of fathers			
Live at home	24.7	39.2	61.0
Live away from home	25.5	37.5	39.0
Residence of mothers			
Live at home	24.4	38.1	86.9
Live away from home	27.9	41.6	13.1
Boarding status			
Does not board	23.2		61.4
Boards	27.5		38.6
Lunch			
Eats at home or brings lunch from home	23.6		54.0
Eats at school cafeteria	26.4		46.0

Source of data: Authors' survey. In this table, the anaemia rates were reported based on the cut-off of 115 g/L for children aged 11 years and under and a cut-off of 120 g/L for children aged 12 years and over. The WHO recommends a haemoglobin level cut-off of 115 g/L for children aged 5-11 years and that of 120 g/L for children aged 12-14 years. The majority of the study children were aged 9-12 years.

WHO=World Health Organization

Parental employment—especially that of the father—also correlated with anaemia rates among children (Table 2). Children whose fathers were involved in off-farm labour had lower anaemia rates than those whose fathers were full-time farmers. While the causality of this relationship and the mechanisms behind it cannot be determined from this analysis, one possible explanation is that parental employment is acting as a proxy for socioeconomic status, which is known to be inversely associated with anaemia and general nutritional status of children (36,37). In China, income from farming is lower than off-farm income (38), and as such, full-time farmers likely have lower household incomes than part-time farmers or off-farm workers.

In terms of school-based characteristics, our data indicate that students who lived in the school dormitories had higher anaemia rates than students who lived at home. Furthermore, students who ate in the school cafeteria had higher anaemia rates than students who ate at home or bought lunches from home (Table 2).

**Table 3.** Individual-, householdand school-based correlates of haemoglobin levels

Dependent variable	Haemoglobin levels (g/L)			
	(1)	(2)	(3)	(4)
Boarding status (0=non-boarder, 1=boarder)	-1.21	-1.42	-1.47	-1.02
	(-2.65)***	(-2.57)**	(-2.65)***	(-1.69)**
Lunch (0=lunch prepared at home, 1=lunch prepared at school)		0.37	0.34	0.45
	(0.68)	(0.63)	(0.76)
Age (months) of students			0.03	0.04
		(2.36)**	(2.80)***
Gender (0=male, 1=female)			-0.61	-0.9
		(-1.35)	(-1.86)**
Education of fathers (0=illiterate, 1=primary school)				-0.3
			(-0.45)
Education of fathers (0=illiterate, 1=junior high school)				1.28
			(1.74)**
Education of fathers (0=illiterate, 1= senior high school)				2.57
			(2.44)**
Education of fathers (0=illiterate, 1= college or above)				-0.4
			(-0.17)
Employment of fathers (0=full-time farmer, 1=part-time farmer)				1.16
			(1.98)**
Employment of fathers (0=full-time farmer, 1=off-farm worker)				2.34
			(2.82)***
Education of mothers (0=illiterate, 1=primary school)				-0.2
			(-0.37)
Education of mothers (0=illiterate, 1=junior high school)				1.48
			(1.91)**
Education of mothers (0=illiterate, 1=senior high school or above)				0.43
			(0.30)
Employment of mothers (0=full-time farmer, 1=part-time farmer)				-0.47
			(-0.82)
Employment of mothers (0=full-time farmer, 1=off-farm worker)				-1.5
			(-1.77)**
Residence of fathers (0=live at home, 1=live away from home)				0.98
			(1.85)**
Residence of mothers (0=live at home, 1=live away from home)				-0.8
			(-1.05)
Constant	125.64	125.55	122.23	120.85
(441.7)***	(402.3)***	(79.05)***	(57.75)***
Observations	4130	4130	4130	4130
R 2	0.002	0.002	0.002	0.01

Source of data: Authors' survey. Figures in parentheses indicate *t* statistics.

***p<0.01;

**p<0.05;

*p<0.1

To examine the relationship more rigorously between the anaemia status and the individual-, householdand school-based factors (Table 3) described above, we conducted a multivariate linear regression (Table 3) using the following specification:

Y_ij_ = a_0_ + a_1_ * Boarding_ij_ + e_ij_ [2]

where Y_ij_ is the Hb level for student *i* in school *j,* and Boarding_ij_ is a dummy variable that is equal to one if student *i* is boarding in school *j*.

As many non-boarding students still ate lunch at school, in equation [3], we controlled for the heterogeneous effects of students who ate lunch at school. The model is:

Y_ij_ = a_0_ + a_1_ * Boarding_ij_ + a_2_ * Lunch_ij_ + e_ij_ [3]

where Lunch_ij_ is a dummy variable that is equal to one if student *i* ate lunch at school *j*. Motivated by the descriptive statistics and the differences that seemed to exist between different gender and age-groups, in equation [4] we controlled for the individual-based characteristics:

Y_ij_ = a_0_ + a_1_ * Boarding_ij_ + a_2_ * Lunch_ij_ + a_3_ * Z_student_ij_ + e_ij_ [4]

where Z_student_ij_ represents a set of controls for age and gender. In our final model, we also controlled for the parental characteristics:

Y_ij_ = a_0_ + a_1_ * Boarding_ij_ + a_2_ * Lunch_ij_ + a_3_ * Z_student_ij_ + a_4_ * Z_parent_ij_ + e_ij_ [5]

where Z_parent_ij_ represents a series of controls for parental education, employment, and residency.

Consistent with the descriptive analysis, the results of multivariate analyses showed a significant correlation between the boarding status and the Hb levels of the students (Table 3). A number of other results from our descriptive analysis were also confirmed, including a significant positive correlation between age and Hb levels. The data also showed that children of fathers with a junior or senior high school education had significantly higher Hb levels than other children. Moreover, paternal employment also mattered: children of fathers who worked either part-time or full-time off the farm had significantly higher Hb levels than children of fathers who were full-time farmers.

### Correlations between anaemia and tests of physical, psychological and cognitive outcomes

Having showing the prevalence of anaemia in the study areas and identifying the factors that significantly correlated with the anaemia status, we examined the relationship between the anaemia status and the performance of students on tests of the physical, psychological and cognitive outcomes. According to our data, anaemia strongly correlated with heights and weights of the students (Table 4). Although the BMI z-scores of all the students were below normal, students with anaemia (n=1,027) had lower BMI than non-anaemic students (n=3,103). Furthermore, only 14.0% of the non-anaemic students had stunted growth (as determined by a height-for-age z-score under −2.0) compared to 27.4% of the anaemic students. Thus, in this population, anaemia was associated with students who had impaired physiological development.

Through the Zhou MHT psychological test, we could measure by self-report how students were able to cope with different stressors (Table 4). The anaemic students had the worse average test scores than the non-anaemic students, suggesting a possible link between anaemia and mental health.

Students with anaemia also performed worse than students without anaemia on the standardized mathematics test. In both fourth and fifth grades, the non-anaemic students, on average, scored above the mean while the anaemic students scored below the mean. Descriptive statistics indicate a difference of 0.54 standard deviation for the fourth grade students and of 0.42 standard deviation for the fifth grade students.

However, as shown in the previous section, there were many correlates of anaemia, and multiple regression was, thus, required to assess the causal effect of anaemia on academic and cognitive performance. To examine the correlation between the anaemia status and these performance measures, we conducted a set of multivariate regressions (Table 5), based on the following model:

Y_ij_ = a_0_ + a_1_ * Anaemia_ij_ + a_2_ * Boarding_ij_ + a_3_ * Lunch_ij_ + a_4_ * Z_student_ij_ + a_5_ * Z_parent_ij_ + e_ij_ [6]

where Y_ij_ can be the standardized mathematics test score, the psychological health test score, or physical outcome measures (height-for-age z-score, stunting status. and BMI); Anaemia_ij_ is a dummy variable equal to one if the child is anaemic according to the age-specific and altitude-adjusted Hb levels; Boarding_ij_ is a dummy variable equal to one if the student boards at school; Lunch_ij_ is a dummy variable equal to one if the student eats lunch at school; Z_student_ij_ represents a set of controls for age and gender; and Z_parent_ij_ represents a series of controls for parental education, employment, and residency.

We found that anaemia significantly correlated with reduced scores on all measures of physical, psychological and cognitive performance. This finding supports the work of another study of over 40,000 school-age children in Gansu province, which found academic achievement (measured by normalized test scores in Chinese, mathematics, and science) to be negatively affected by iron deficiency (20). Our findings are consistent with a hypothesis that the rash of anaemia in rural Qinghai and Ningxia schools may, in part, underlie the reduced academic achievement of rural students.

**Table 4.** Physical, psychological and cognitive test scores, by anaemia status

Correlate	Anaemic students	Non-anaemic students
Body mass index (z-score) *	-0.64	-0.49
Height-for-age (z-score) **	-1.12	-0.55
% with stunted growth	27.4	14.0
Aggregate mental health test (max=90, where lower scores indicate better mental health)	41.7	39.3
Grade 4 mathematics test—distance from mean (standardized test score, with mean=0 and standard deviation=1)	-0.39	0.15
Grade 5 mathematics test—distance from mean (standardized test score, with mean=0 and standard deviation=1)	-0.33	0.09

Source of data: Authors' survey. In this table, the anaemia rates were reported based on the cut-off of 115 g/L for children aged 11 years and under and a cut-off of 120 g/L for children aged 12 years and over. The WHO recommends a haemoglobin level cut-off of 115 g/L for children aged 5-11 years and that of 120 g/L for children aged 12-14 years. The majority of the sample population were aged 9-12 years.

*The BMI z-scores (kg/m ^2^) were defined based on the WHO reference 2007 for both the genders aged 5-19 years (39,40);

**HAZ z-scores (height in cm) were defined based on the WHO reference 2007 for both the genders aged 5-19 years (41-42). In this paper, stunting was defined based on the WHO reference 2007 for HAZ, where any student with a HAZ of lower than −2 was considered to be stunted.

HAZ=Height-for-age z-score;

WHO=World Health Organization

## DISCUSSION

We have shown that anaemia is widespread in rural Qinghai and Ningxia elementary schools. The overall rate of anaemia was 24.9%, with the large regional variation, likely due to the differences in income and the existence of distinct minority groups in certain areas. Both of these (and related) factors are related to differences in diet and lifestyle that may affect the levels of anaemia. Despite the regional variation, none of the 76 schools in our sample had an average rate of anaemia below 5%, indicating that, according to the WHO classification, the prevalence of anaemia in all the sample schools is high enough to be considered a public-health problem. Since our sample region is representative of the poor, rural regions in Ningxia and Qinghai, our results imply that hundreds of thousands of children in these two regions alone may be anaemic. Yet, during an earlier survey, when asked about whether anaemia was a problem among their students, over 90% of principals believed that it was not, or simply did not know (23). Iron deficiency remains a significant nutrition issue in poor rural northwest China, which we hope will be addressed by policy-makers and educators.

**Table 5.** Correlations between anaemia and physical, psychological, and cognitive outcomes

Dependent variable	Standardized mathematics test score (mean=0, SD=1)	Psychological test score (maximum=90)	Height-forage z-score	Student is stunted (0=no, 1=yes)	BMI z-score
Anaemia status (0=not anaemic, 1=anaemic)	-0.39	1.75	-0.40	0.11	-0.09
	(-10.54)***	(3.52)***	(-8.52)***	(7.81)***	(-2.43)**
Boarding status (0=non-boarder, 1=boarder)	-0.14	1.38	0.055	0.0034	0.14
	(-3.50)***	(2.62)***	(1.11)	(0.23)	(3.40)***
School lunch (0=eats lunch prepared at home, 1=eats school lunch)	0.01	-0.37	0.02	-0.01	0.07
	(0.19)	(-0.71)	(0.51)	(-0.52)	(1.89)**
Age (months) of students	-0.002	0.01	-0.05	0.01	-0.02
	(-2.33)**	-1.06	(-46.1)***	(23.4)***	(-17.3)***
Gender (0=male, 1=female)	-0.07	1.54	0.03	-0.002	-0.06
	(-2.20)**	(3.63)***	(0.83)	(-0.21)	(-1.81)**
Education of fathers (0=illiterate, 1=primary school)	-0.05	0.16	-0.07	0.03	-0.05
	(-1.03)	(0.27)	(-1.33)	(2.04)**	(-1.05)
Education of fathers (0=illiterate, 1=junior high school)	0.09	-0.52	-0.19	0.02	-0.13
	(1.94)**	(-0.80)	(-3.19)***	(1.34)	(-2.56)**
Education of fathers (0=illiterate, 1=senior high school)	0.23	-0.32	-0.18	0.004	-0.03
	(3.32)***	(-0.35)	(-2.05)**	(0.15)	(-0.48)
Education of fathers (0=illiterate, 1=college or above)	-0.16	2.19	-0.38	0.02	-0.06
	(-1.01)	(1.06)	(-1.98)**	(0.29)	(-0.35)
Employment of fathers (0=full-time farmer, 1=part-time farmer)	0.11	-0.15	-0.02	-0.01	0.01
	(2.74)***	(-0.29)	(-0.46)	(-0.54)	(0.16)
Employment of fathers (0=full-time farmer, 1=off-farm worker)	0.08	0.24	0.19	-0.05	0.1
	(1.43)	(0.32)	(2.81)***	(-2.49)**	(1.80)*
Education of mothers (0=illiterate, 1=primary school)	0.08	-0.79	-0.002	0.01	0.06
	(2.18)**	(-1.64)	(-0.04)	(-0.48)	(1.53)
Education of mothers (0=illiterate, 1=junior high school)	0.26	-2.41	-0.02	-0.02	0.07
	(5.15)***	(-3.55)***	(-0.27)	(-1.01)	(1.33)
Education of mothers (0=illiterate, 1=senior high school or above)	0.2	-2.98	0.02	-0.02	0.17
	(2.10)**	(-2.36)**	(0.13)	(-0.50)	(1.73)*
Employment of mothers (0=full-time farmer, 1=part-time farmer)	-0.06	-0.03	0.09	-0.003	0.01
(-1.61)	(-0.06)	(1.93)*	(-0.19)	(0.20)
Employment of mothers (0=full-time farmer, 1=off-farm worker)	-0.07	0.54	-0.07	0.03	-0.05
	(-1.19)	(0.73)	(-1.05)	(1.67)*	(-0.95)
Residence of fathers (0=live at home, 1=live away from home)	0.19	-0.73	0.07	-0.01	-0.01
	(-1.57)	(1.58)	(-0.76)	(-0.40)	(5.50)***
Residence of mothers (0=live at home, 1=live away from home)	-0.07	0.6	-0.03	0.02	-0.09
	(-1.41)	(0.89)	(-0.44)	(1.10)	(-1.70)*
Constant	0.31	38.52	5.65	-0.82	1.48
		(2.19)**	(19.96)***	(31.15)***	(-15.20)***	(9.85)***
Observations	4130	4130	4130	4130	4130
R 2	0.08	0.02	0.41	0.17	0.09

Source of data: Authors' survey. Figures in parentheses indicate *t* statistics. In this table, the anaemia rates were calculated based on the cut-off of 115 g/L for children aged 11 years and under and that of 120 g/L for children aged 12 years and over. The WHO recommends a haemoglobin level cut-off of 115 g/L for children aged 5-11 years and that of 120 g/L for children aged 12-14 years. The majority of the sample children were aged 9-12 years.

***p<0.01;

**p<0.05;

*p<0.1;

BMI=Body mass index;

SD=Standard deviation;

WHO=World Health Organization

We have also identified several factors that are correlated with anaemia status. Students who lived and ate at school had higher rates of anaemia. Children with parents who were less educated or who worked in the farm were more likely to be anaemic.

These findings are consistent with results of our previous work, in which we have shown that boarding schools in rural Shaanxi province are ill-equipped to deliver sufficient nutritional content in school lunches to their students (25). Results of the study indicate that 23% of students who boarded were stunted compared to only 11% of students who lived at home.

We also found that the anaemia status negatively correlated with the performance on tests of physical and cognitive development. Children with anaemia were shorter for their age, and a higher percentage of them had stunted growth. The anaemia status significantly correlated with lowered academic achievement as measured by a standardized mathematics examination.

Since this study was built on the work of a previous survey in Shaanxi province in 2008, it is useful to compare the results between the two surveys. Both the surveys found high rates of anaemia in the survey areas, although these rates were slightly higher in Shaanxi. Both the surveys found strong links between the Hb levels and both boarding status and parental education. Both the surveys found a strong negative relationship between the anaemia status and the performance on tests of physical, mental, and cognitive well-being, although this relationship was more pronounced in Ningxia and Qinghai. This paper went beyond the Shaanxi paper in its findings of a strong link between parental employment and Hb levels.

Although we were not able to pinpoint the exact determinants of anaemia, the main implication of this work is that anaemia remains a serious health problem among children in rural Qinghai and Ningxia. However, none of the correlates we measured explains the full extent of the anaemia problem, which suggests fundamental determinants we did not measure. Furthermore, our correlative analysis did not allow us to determine causality. It does raise significant issues such that more research in this area is needed.

The findings of the present study and those in other recent work by us and others Showed that anaemia rates in elementary schools in the poor, rural areas of China are high and widespread. Given the known consequences of anaemia—on health, educational performance, and behaviour—, it seems that China's education (and health) officials should take serious steps to alleviate the problem. In recent years, the Ministry of Education has spent millions of dollar on new facilities and teachers. There is no doubt that the school merger programme has resulted in better schools. However, as observed in this study, the high rates of anaemia—especially among the boarding school students—mean that these students are almost certainly unable to take full advantage of the improvements in rural elementary education. We believe that the results of our study demand continued research on the causes and consequences (and possible solutions) of anaemia in rural schools across China. Among other things, experimenting with different ways of improving iron intake among boarding children in western China seems warranted.

## ACKNOWLEDGEMENTS

The authors acknowledge the financial assistance of the National Natural Science Foundation of China (70903064), the Institution of Geographic Sciences and Natural Resources Research, Chinese Academy of Sciences (200905007), and the Ford Foundation.

**Appendix Table 1.** Altitude and unadjusted haemoglobin levels

Item	Altitude (metres)	Unadjusted haemoglobin level (g/L) *	% of students with anaemia †	Size of Hb adjustment due to altitude$
Total sample	1,842	131.9	10.7	-6.7
By province				
Qinghai	2,344	129.9	14.8	-11.0
Ningxia	1,566	133.0	8.4	-4.4
By county				
Qinghai province			
Xunhua ( 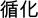 )	2,485	120.8	34.1	-12.5
Ledu ( 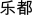 )	2,171	136.5	3.2	-9.0
Hualong 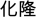 )	2,469	130.1	8.1	-12.2
Huzhu ( 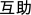 )	2,529	135.3	6.3	-12.7
Minhe ( 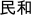 )	1,981	134.9	5.4	-7.4
Ningxia province			
Yanchi ( 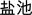 )	1,343	128.5	11.2	-2.9
Tongxin ( 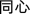 )	1,349	127.4	14.8	-3.0
Haiyuan ( 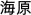 )	1,787	138.6	2.6	-5.9
Longde ( 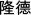 )	1,645	135.7	6.9	-4.8
Jingyuan ( 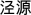 )	1,623	130.5	8.9	-4.6

Source of data: Author's survey:

*In this table, the anaemia rates were calculated based on the cut-off of 115 g/L for children aged 11 years and under and that of 120 g/L for children aged 12 years and over. The WHO recommends a haemoglobin level cut-off of 115 g/L for children aged 5-11 years and that of 120 g/L for children aged 12-14 years. The majority of the sample population were aged 9-12 years.

† The 10.7% figure represents the average anaemia rate using the haemoglobin level without adjustment of altitude;

$ The difference of haemoglobin levels with and without account for altitude effects according to calculations by U.S. CDC (28).

CDC=Centers for Disease Control and Prevention;

Hb=Haemoglobin;

WHO=World Health Organization
